# SARS-CoV-2 Molecular Transmission Clusters and Containment Measures in Ten European Regions during the First Pandemic Wave

**DOI:** 10.3390/life11030219

**Published:** 2021-03-09

**Authors:** Maria Bousali, Aristea Dimadi, Evangelia-Georgia Kostaki, Sotirios Tsiodras, Georgios K. Nikolopoulos, Dionyssios N. Sgouras, Gkikas Magiorkinis, George Papatheodoridis, Vasiliki Pogka, Giota Lourida, Aikaterini Argyraki, Emmanouil Angelakis, George Sourvinos, Apostolos Beloukas, Dimitrios Paraskevis, Timokratis Karamitros

**Affiliations:** 1 Bioinformatics and Applied Genomics Unit, Department of Microbiology, Hellenic Pasteur Institute, 11521 Athens, Greece; mbousali@gmail.com (M.B.); aristeadimadi@gmail.com (A.D.); vpoga@pasteur.gr (V.P.); 2 Department of Hygiene Epidemiology and Medical Statistics, School of Medicine, National and Kapodistrian University of Athens, 15772 Athens, Greece; ekostakh@med.uoa.gr (E.-G.K.); gmagi@med.uoa.gr (G.M.); 3 4th Department of Internal Medicine & Infectious Diseases, School of Medicine, National and Kapodistrian University of Athens, 15772 Athens, Greece; tsiodras@med.uoa.gr; 4 Medical School, University of Cyprus, 2029 Nicosia, Cyprus; nikolopoulos.georgios@ucy.ac.cy; 5 Laboratory of Medical Microbiology, Department of Microbiology, Hellenic Pasteur Institute, 11521 Athens, Greece; sgouras@pasteur.gr (D.N.S.); e.angelakis@pasteur.gr (E.A.); 6 IRD, APHM, VITROME, IHU-Mediterranean Infections, Aix Marseille University, 13005 Marseille, France; 7 Department of Gastroenterology, Medical School of National and Kapodistrian University of Athens, “Laiko” General Hospital of Athens, 11527 Athens, Greece; gepapath@med.uoa.gr; 8 Infectious Diseases Clinic A, Sotiria Chest Diseases Hospital, 11527 Athens, Greece; giotalourida@gmail.com (G.L.); katrin.argyraki@gmail.com (A.A.); 9 Laboratory of Clinical Virology, School of Medicine, University of Crete, 71500 Heraklion, Greece; sourvino@med.uoc.gr; 10 Department of Biomedical Sciences, University of West Attica, 12243 Athens, Greece; 11 Institute of Infection and Global Health, University of Liverpool, Liverpool L69 7BE, UK

**Keywords:** SARS-CoV-2, COVID-19, pandemic, transmission, clusters, phylodynamics, phylogenetics, molecular epidemiology, molecular transmission clusters

## Abstract

Background: The spatiotemporal profiling of molecular transmission clusters (MTCs) using viral genomic data can effectively identify transmission networks in order to inform public health actions targeting SARS-CoV-2 spread. Methods: We used whole genome SARS-CoV-2 sequences derived from ten European regions belonging to eight countries to perform phylogenetic and phylodynamic analysis. We developed dedicated bioinformatics pipelines to identify regional MTCs and to assess demographic factors potentially associated with their formation. Results: The total number and the scale of MTCs varied from small household clusters identified in all regions, to a super-spreading event found in Uusimaa-FI. Specific age groups were more likely to belong to MTCs in different regions. The clustered sequences referring to the age groups 50–100 years old (y.o.) were increased in all regions two weeks after the establishment of the lockdown, while those referring to the age group 0–19 y.o. decreased only in those regions where schools’ closure was combined with a lockdown. Conclusions: The spatiotemporal profiling of the SARS-CoV-2 MTCs can be a useful tool to monitor the effectiveness of the interventions and to reveal cryptic transmissions that have not been identified through contact tracing.

## 1. Introduction

Coronaviruses (CoVs) belong to the Nidovirales order, Coronaviridae family and Coronavirinae subfamily and are the largest known group of viruses. Within the past two decades, two newly emerged coronaviruses, Severe Acute Respiratory Syndrome Coronavirus (SARS-CoV) and Middle East Respiratory Syndrome Coronavirus (MERS-CoV), have caused serious respiratory and intestinal infections in humans [1]. In December 2019, a novel Coronavirus was firstly reported in the city of Wuhan, Hubei province in China. This novel coronavirus was further named as “2019-nCoV” by WHO [2] and later as Severe Acute Respiratory Syndrome Coronavirus 2 (SARS-CoV-2) by the International Committee on Taxonomy of Viruses. On 24 January 2020, the first European case was reported in France while four days later, Germany confirmed its first case. After six days, on 30 January 2020, WHO declared the 2019-nCoV outbreak to be a Public Health Emergency of International Concern (PHEIC) under International Health Regulations. As of 10 January 2021, almost 88.4 million infections were recorded worldwide, including 1.9 million deaths (https://www.who.int/publications/m/item/weekly-epidemiological-update---12-January-2021, accessed on 16 October 2020). As soon as, the 11 January 2020, the first whole-genome sequence of SARS-CoV-2 was available and became the baseline for researchers to track SARS-CoV-2, as it spread through the world [3]. Based on phylogenetic studies, SARS-CoV-2 belongs to the same lineage as SARS-CoV and MERS-CoV [4,5,6]. An unprecedented number of full genome sequences have become available thanks to the worldwide effort of scientists and to the GISAID consortium [7].

SARS-CoV-2 exhibits a high potential to undergo human-to-human transmission, while the three major factors that are involved in spreading, are the source of infection, the route of transmission and population susceptibility coupled with viral latency [8,9]. The primary transmission mode is person-to-person contact through respiratory droplets, as well as direct contact with an infected subject or indirect contact, through hand-mediated transfer of the virus from contaminated fomites to the mouth, nose, or eyes. The novel coronavirus SARS-CoV-2 is less deadly, but far more transmissible than MERS-CoV or SARS [10], while it has been found that transmission of SARS-CoV-2 is mostly driven by clusters in close contacts, particularly family clusters, and less so by community transmission (https://www.who.int/docs/default-source/coronaviruse/who-china-joint-mission-on-covid-19-final-report.pdf, accessed on 16 October 2020) although super-spreading events continue to occur in the pandemic. 

Based on up to date data, there are seven clades of SARS-CoV-2 in total, denoted as G, GH, GR, L, O, S, and V) [11], while the evolutionary rate has been estimated at approximately 10^−3^–10^−4^ substitutions per site per year [12,13,14,15,16], which is broadly in line with those estimated for SARS-CoV [17] and Middle East respiratory syndrome [18], about a third of that estimated for influenza B and in general lower than other RNA viruses [19,20]. Phylogenetic analysis of SARS-CoV-2 data is challenging, not only because of the magnitude of available data, but also because of the way the virus has spread in the population in such a short period [21]. As a result, dedicated bioinformatics pipelines and big data analysis tools are of great value in order to rapidly evaluate the factors that are associated with the spread and the transmissibility of the virus. Phylogenomics and phylodynamics analysis in almost real time after the collection and sequencing of the samples may assist in focusing the prevention efforts after the identification of transmission clusters in the communities.

Transmission clusters are groups of infected individuals who are connected with SARS-COV-2 transmission and potentially represent a subset of a risk network. The identification of molecular transmission clusters (MTCs) provides a tool to identify transmission clusters and risk networks and has been previously used for various pathogens, including HIV, Influenza A, and *Mycobacterium tuberculosis* [22,23,24,25,26]. A time-space cluster occurs by focusing on the identification of MTCs in a particular geographic area while using time-reversible phylodynamics approaches. MTCs have been used to examine the likely impact of genetic (mutations and subtypes of viruses) [27,28,29], demographic and clinical [22,25,26] factors on regional phylogenetic clustering. Identification of MTCs for HIV has resulted in the characterization of the risk factors that are associated with the spread of the virus in different countries [22,30,31]. As for SARS-CoV-2, an analysis of 3184 sequences from Japan with complete metadata, revealed that the primary source of clusters are healthcare facilities such as hospitals and care facilities such as nursing homes [32].

Outbreak containment through testing, case isolation, contact tracing, and quarantine is often the first line of defense against a novel emerging infectious disease [33,34]. During the first pandemic wave of SARS-CoV-2, efforts to contain the outbreak of the virus failed and in order to flatten the epidemic curve supplemental containment measures were taken in almost all countries worldwide [35]. These measures rely on the capacity to control viral transmission from person-to-person and their prioritization can be determined by understanding the virus’ transmission patterns [36]. In Europe, the containment measures taken included large-scale physical distancing measures and movement restrictions—stay at home orders often referred as lockdowns, non-essential shop closure, national events stop, school, nurseries, kindergartens and educational facilities closure, and national and international movement and flights restrictions [37,38,39]. Containment measures may have different efficacy in diverse age groups [40], and the groups for which the measures are more effective may vary across populations [41]. Moreover, demographic, socioeconomic, climatic and cultural factors have been linked to the distribution of COVID-19 cases across the world and they have a potential impact on the effectiveness of the containment measures, too [42]. 

In this study, we developed dedicated bioinformatics pipelines to conduct a massive identification and spatiotemporal characterization of SARS-CoV-2 MTCs in 10 European geographical regions. We also examine the likely impact of demographic and epidemiological factors, as well as the effect of the containment measures taken, on the regional phylogenetic clustering of the pandemic.

## 2. Materials and Methods

### 2.1. Raw Data Pre-Processing and Filtering

All of the available SARS-CoV-2 sequencing data and metadata derived from European samples were downloaded from GISAID [7] on 16 October 2020. From the 82,572 raw sequences originated from the European continent, 5352 sequences were removed from the dataset as they were either incomplete (<29,000 bp) or of low genomic coverage (sequences containing >5% Ns), resulting in 77,220 sequences originated from 1376 different European geographical regions, in total. In order to ensure a maximum genetic diversity, a threshold was set in order to keep only those geographical regions with more than 80 sequences available. Geographical regions were further filtered based on the existence of more than three different SARS-CoV-2 clades spread in the population as well as on the continuity of samples for a minimum period of four weeks after the establishment or after the end of a containment measure. The geographical regions that met all of the above-mentioned criteria were Munich (DE), La Rioja (ES), Navarra (ES), Madrid (ES), Liege (BE), Vienna (AT), Lombardy (IT), Reykjavik (IS), Uusimaa (FI), and Saint Petersburg (RU).

### 2.2. Reconstruction of Phylogenetic Trees and Phylodynamics Analysis

For each geographical region a separate phylogenetic and phylodynamics analysis was undertaken in order to perform spatiotemporal characterization of MTCs. Firstly, each geographical region’s data and metadata were joined with world’s formatted data and metadata and a FASTA formatted file was generated (sequences.fasta) as well as a TSV file with the corresponding metadata (metadata.tsv). Those files were used as input in Nextstrain’s “augur” pipeline [43] after excluding duplicates. The pipeline involves sequence alignment with MAFFT [44] using NC_045512.2 (also referred to as SARS-CoV-2 isolate Wuhan-Hu-1 GenBank ID: MN908947.3) as the reference sequence and a subsequent subsampling step of sequences that are not referring to the geographical region of interest, based on genetic (alignment), time (collection date) and space (region of exposure) criteria. The NC_045512.2 sequence, was also used during the phylogeny reconstruction along with “Wuhan/WH01/2019” sequence (MT291826.1). The sub-sampled sequences were filtered/processed in parallel with the study sequences. The phylogenetic tree reconstruction was performed using IQ-TREE [45] and the general time-reversible substitution model with gamma rate heterogeneity among sites (GTR+G substitution model), as suggested by “ModelFinder” (implemented in IQ-TREE) [46], and as described in previous studies on SARS-CoV-2 phylogeny [47,48,49]. Re-rooting, resolving of polytomies, inferring of internal node dates, labeling of internal nodes, pruning of sequences and maximum-likelihood phylodynamics analysis was performed with TreeTime [50] using the default parameters that are pre-optimized for SARS-CoV-2 by Nextstrain.

### 2.3. Identification of Molecular Transmission Clusters (MTCs)

In order to identify MTCs, a two-step approach was followed as previously described by Paraskevis et al. [22]. Given the rapid spread in the population and the relatively slow mutation rate of SARS-CoV-2, which results in low overall genetic variability [51,52], the identification of MTCs from the generated phylogenetic trees was performed using two different criteria: clusters with maximum genetic distance ≤0.005 [16,53] as well as Shimodaira–Hasegawa support (SH-Test) ≥0.75 [54] (phylogenetic confidence criterion) and clusters consisting of geographical region’s specific sequences at a proportion of >65% compared to the total number of sequences within the cluster (geographic criterion). The filtering of MTCs in the trees was performed using in-house developed scripts written in R programming language v4.0.3 (https://www.R-project.org/, accessed on 16 October 2020), utilizing the “ape” [55] and the “phangorn” [56] libraries for phylogenetics and evolutionary analysis, the tidyverse R package for data handling and the “ggtree” library for the visualization of the phylogenies [57] as well as Bash sh-compatible command language. 

### 2.4. Statistical Analysis

All variables were examined as categorical. The continuous variables of age and collection date that were retrieved from GISAID, were transformed into categorical. More specifically, we created five age groups based on social activity (0–19, 20–34, 35–49, 50–64, 65+ years old) as described by Pollan et al. [58]. As for the collection date, we created 32 classes that are referring to weeks, starting from 2020-02-20 when the first sample in the dataset was collected. For simple comparisons of distributions at different levels of categorical variables we performed pairwise chi-square tests, as well as Fisher’s exact test, while the statistical significance threshold was set at 5% (*p*-value < 0.05). All calculations were performed using R programming language v4.0.3 (https://www.R-project.org/, accessed on 16 October 2020).

## 3. Results

### 3.1. Phylogenetic Analysis and Characterization of Molecular Transmission Clusters

**Figure 1:**
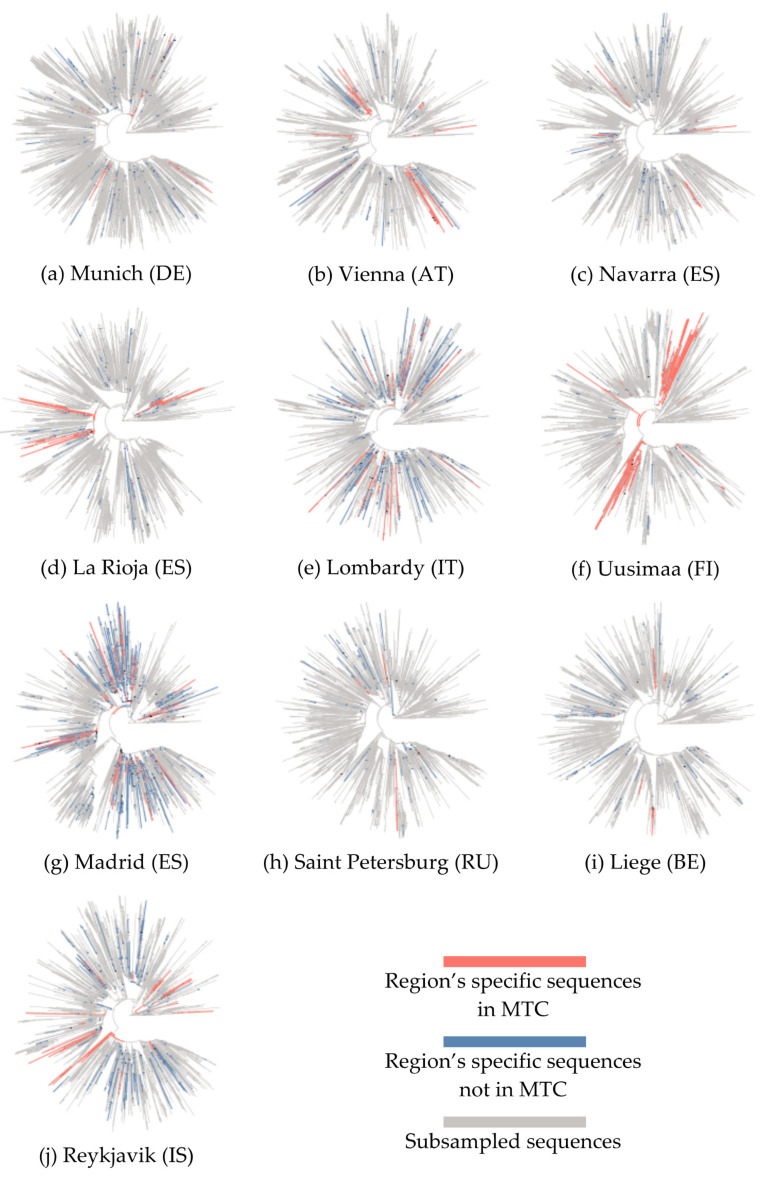
Rooted phylogenetic inference of full genome SARS-CoV-2 sequences from 10 European geographical regions (**a**–**j**) and a globally sampled dataset (in grey). Nodes significantly enriched in geographical region’s specific sequences representing molecular transmission clusters (MTCs) are colored in orange, while nodes with geographical region’s specific unclustered sequences are in blue.

For each geographical region a phylogenetic tree was reconstructed using the filtered sequence data of the geographical region and the sub-sampled sequences through the world (Figure 1). Reykjavik, Madrid and Liege (Figure 1g,i,j) account for more than 500 geographical region’s specific sequences while Munich, Vienna, and Navarra (Figure 1a–c) account for less than 200 sequences. After the subsampling, however, all trees were generated with more than 2950 sequences (Table 1). The collection dates in our dataset ranged as the first sequences were reported in Lombardy on 2020-02-20 while the last ones were from Liege on 2020-09-25. Madrid and Lombardy were found to have the most MTCs (20 and 14 respectively), while the other geographical regions had less than 10 MTCs. At the same time, the percentage of clustered sequences was higher in Uusimaa and La Rioja (88.9% and 67.5%, respectively), while Saint Petersburg and Liege were found to have high percentage of sequences not belonging to MTCs (unclustered). Large community clusters and were observed in all geographical regions with the exception of Saint Petersburg. Especially in Uusimaa, a super-spreading event was found (>20 geographical region’s specific sequences [59]), possibly occurred as an MTC consisting of 156 sequences. Smaller clusters were found in all geographical regions. Moreover, patterns based on the clade of the clustered sequences were observed. All geographical regions had great proportion of clustered sequences belonging to the GR clade, except for the Spanish geographical regions of Madrid, La Rioja as well as Uusimaa for which the GR clade was present only in unclustered sequences (25 in Madrid, 3 in La Rioja, and 6 in Uusimaa). In general, geographical regions with fewer clustered sequences showed greater diversity in the distribution of clades, while the combination of distributions of O and V clades were rare and were observed only in Reykjavik.

### 3.2. Demographic Characteristics and Molecular Transmission Clusters

**Figure 2:**
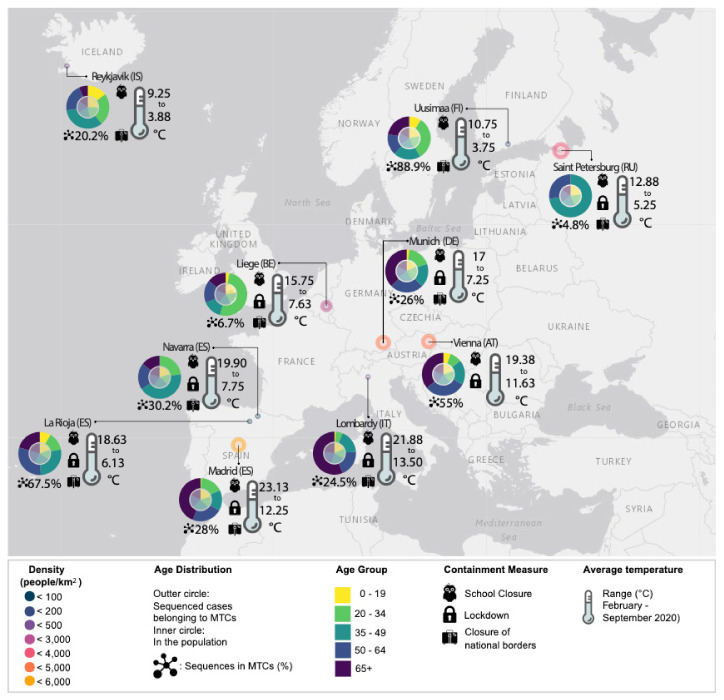
Distribution of total sequenced cases (five-color inner circle) and clustered sequences (belonging to MTCs—outer circle) according to age group. Density of population, containment measures, total levels of clustering and averages temperature range for each geographical region are also presented.

We investigated the hypothesis that cases belonging to particular age groups might be more important contributors in the formation of MTCs, as their social activity profiles differ substantially. Interestingly, as presented in Table 2, specific age groups were more likely to belong to MTCs in different regions. In detail, in Reykjavik, cases referring to the age group 0–19 were more likely to belong to MTCs (*p* ≤ 0.05). In Liege, cases referring to the age group 20–34 were more likely to belong to MTCs (*p* ≤ 0.05) while in Saint Petersburg cases referring to the age group 35–49 were found more likely to belong to MTCs (*p* < 0.01). Although no specific age groups were directly associated with molecular clustering across all regions analyzed, it is of great interest that these three particular regions presented the lower levels of total clustering, with only 4.8%, 6.7%, and 20.2% of total sequences in MTCs for Saint Petersburg, Liege and Reykjavik, respectively (Figure 2, outer five-color circles). Simultaneously, the age groups of the sampled sequences were evenly distributed in these regions (Figure 2. inner five-color circles), indicating no sampling bias. These findings suggest that in settings with lower levels of clustered dispersal, younger (<50 y.o.) and consequently socially more active individuals drive the formation of MTCs. As expected, gender was not found to be associated with the formation of MTCs in none of the geographical regions studied.

### 3.3. Containment Measures Taken and Molecular Transmission Clusters

**Figure 3:**
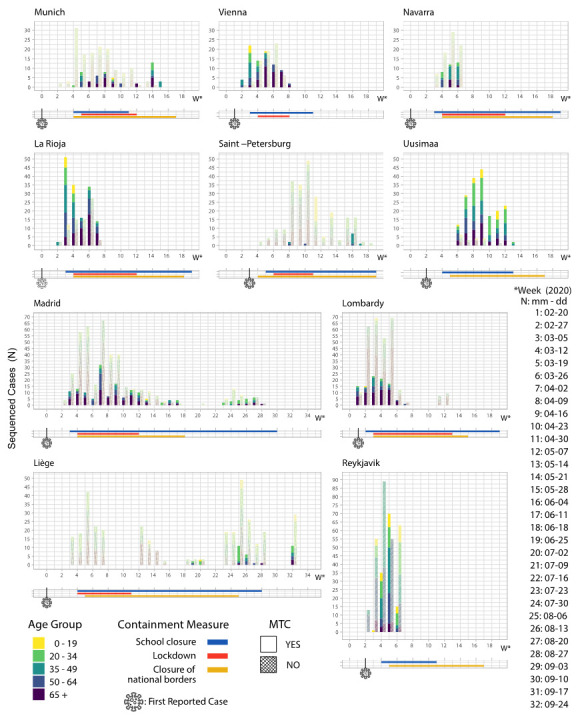
Distribution of clustered and unclustered sequences for each age group during the containment measures of schools’ closure (blue), the lockdown (red) and closure of national borders (orange). The virus symbol refers to the first case reported in each of the geographical regions.

In order to investigate the effect of the containment measures taken on the individual population groups for each geographical region we analyzed the dynamics of both the clustered (in MTCs) and the unclustered sequences on the time scale, with regards to each of the five age groups (0–19, 20–34, 35–49, 50–64, 65+) (Figure 3). In order to examine the effects of each measure in the clustering of each age group, we compared the number of clustered sequences belonging to particular age groups (where available) before the establishment of a measure and one/two weeks after the establishment—or the end—of the measure, as SARS-CoV-2 has an average incubation period of 6–7 days in average.

We studied the effect of the containment measures in each of the ten European regions on the total number of clusters (MTCs, N), as well as the total number of sequences that were found to be clustered (Number of sequences in each MTC). In all regions that chose lockdown as a containment measure, we observed an increase of the clustered sequences referring to the age groups 50–64 and 65+, two weeks after the establishment of the measure. Especially in Munich, La Rioja, Vienna, Navarra, and Lombardy, MTCs referring to the age group 65+ were de novo formed two or more weeks after the establishment of the measure. At the same time, in Madrid, an increase of MTCs is observed in the age group 50–64. Moreover, in Munich (DE), clustered sequences referring to cases at the age group 20–34 were eliminated two weeks after implementation of movement restrictions, while those referring to age groups 20–34 and 35–49 were increased after the end of this measure (Figure 3).

As for the closure of the schools, in Vienna (AT) and La Rioja (ES), we observed a decrease (75% and 100% decrease, respectively) in the number of pre-existing clustered sequences referring to the age group 0–19 two weeks after the establishment of the measure, indicating a successful containment of the transmissions within this group. MTCs referring to age group 20–34 were also shrunk. The decrease of the MTCs belonging to age group 20–34 in Munich (DE) coincided and may also be associated with schools’ closure, as MTCs were formed one week after school closure, thereby suggesting that the corresponding infections were traced at one week before the establishment of the measure. This trend was not obvious in Uusimaa (FI) and Reykjavik (IS), where the pre-existing clusters of younger infected individuals persisted two weeks after the establishment of the measure (Figure 3). Of note, in these geographical regions, school closure was not combined with a generalized lockdown. 

The national borders closure was generally applied for longer periods and data availability after the end of the measure was limited for most of the geographical regions evaluated in this study. Where available, data from Liege (BE) and Madrid (ES) suggested that after the reopening of the borders the expected increase of the unclustered sequences was followed by new MTCs referring to all age groups and particularly to the age group of 20–34 (100% and 50% increase, respectively), yet excluding young individuals 0–19 y.o. (Figure 3).

## 4. Discussion

In the present study, we focused on the spatio-temporal characterization of SARS-CoV-2 molecular transmission clusters in ten geographical regions of the European continent and we evaluated the effect of individual containment measures taken in each geographical region on the clustering patterns of the sequenced cases and the virus dispersal profiles. One major factor we focused on was the role of the different age groups of the infected individuals as the contribution of this characteristic in the spread of COVID-19 remains unclear [60]. The containment measures we focused on were the lockdowns, the school closures, and border closures, as these measures have different impacts on different parts of the population, but also directly affect all aspects of the society. To this end, each country should undertake all containment measures needed towards protecting health, both physical and mental [61,62,63], while preventing economic and social disruption and respecting human rights (https://www.who.int/director-general/speeches/detail/who-director-general-s-opening-remarks-at-the-media-briefing-on-covid-19---11-march-2020, accessed on 16 October 2020). At certain points, some countries have had no other option but to issue stay-at-home orders in order to buy time and prepare their health-care systems for the pandemic, test the population in a wide-scale and trace and quarantine contacts (https://www.who.int/news-room/q-a-detail/herd-immunity-lockdowns-and-covid-19, accessed on 16 October 2020). 

The social activity profiles of the different age groups are expected to differ. Younger individuals are usually more socially active, thus are expected to be more important contributors in the formation of MTCs [64]. Our findings suggest that this pattern did not apply uniformly across the different settings. Analyzing the distribution of clustered sequences according to the age group they belonged to, we found that younger individuals were more likely to belong to MTCs (Table 2) but only in settings with lower overall levels of total clustering (Saint Petersburg, Liege and Reykjavik) (Figure 2, outer five-color circles). At the same time, the age distribution of the sampled cases in these regions was balanced, as in all regions included in the study (Figure 2, inner five-color circles). Multiple factors could have been associated with the formation of MTCs but by analyzing the demographic and socio-economic characteristics of these as well as of all regions studied, we did not identify other factors that could have driven this trend (Table S1). The fact that especially during the initial phase of the pandemic there was a lot of fear for the elderly—thus they were more protected—could explain the observation that MTCs were primarily formed amongst younger individuals, in these particular settings [65]. 

Across Europe, the kind and the timing of the containment measures taken as a response to the coronavirus pandemic has differed from country to country as presented in detail in Supplementary Figure S1. Among the first measures taken were travel bans from specific locations. This was followed by local or regional quarantines, calls for self-isolation and social distancing. In some countries, extensive closures and quarantines followed. Based on the results of the present study, in most of the geographical regions that established lockdowns during the first pandemic wave, an increase of the MTCs that refer to the older population of the geographical regions evaluated was observed two weeks after the establishment of the measure (age classes 50–64 and 65+ yrs) (Figure 3). However, it is of higher importance to note that sampling bias may apply on this type of analysis and have an impact on the conclusions drawn, especially since different countries may have drastically divergent sampling and sequencing approaches, which may also change over time. 

Taking these limitations into account, our results suggest that the generalized lockdown might be effective in containing the epidemic within the most socially active groups of the population but the impact of this measure on the transmissions associated with older adults warrants further investigation. Similarly, Salazar et al. analyzed the daily number of COVID-19 cases for different age groups before and after the lockdown period, detecting a relative increase in the incidence of detected SARS-CoV-2 infection in age groups 50–54 and 55–59 years, after the initial implementation of the measure [41]. A possible explanation could be the household transmissions that occurred because of the lockdown, especially in geographical regions or areas where most of the households are multi-generational (all Spanish geographical regions and Lombardy in Italy). Another explanation could be the higher employment rates in middle-aged adults compared with the younger or even that younger workforce is more related to employment that could be performed digitally/from home compared to middle-aged population. Adam et al. observed that transmission within family households were more frequent than within social and work settings. They highlighted that social settings were associated with both younger cases and more secondary cases compared to households, although this was not the case for households versus work setting [65]. These trends can only be confirmed by carefully designed controlled studies that specifically measure the changes in the MTCs’ distribution over time.

School closures have been widespread in some countries during influenza pandemics, and many studies report important effects on reducing transmission [66]. Expecting that the benefits observed in influenza outbreaks would also apply to SARS-CoV-2, many countries instituted large-scale or national closure of schools by March, 2020. Previous studies regarding the transmission dynamics and clinical characteristics of SARS-CoV-2 infection in children have suggested that children may become a significant spreader at the explosion stage of the outbreak [66], while at the same time the risk of diffusion of infection from child to child is low and even the transmission from a child to an adult is uncommon [67,68,69]. Moreover, as Piovani et al. suggest [70], combination of mass gathering bans and school closures is associated with the reduction in COVID-19 mortality. In this study, we sought to investigate the effect of school closure in MTCs patterns. Based on our analyses, and for those geographical regions with adequate supporting data, school closure was possibly related to the decrease of clustered sequences referring to the age group 0–19 and/or 20–34 in Vienna (AT), La Rioja (ES), and Munich (DE) (Figure 3). This leads to the hypothesis that clustered spread of the virus was decreased due to the limited interactions of these age groups, but also probably due to limited movements and interactions of young parents taking their children to schools. At the same time, in Uusimaa (FI) and Reykjavik (IS), the pre-existing clusters of younger infected individuals remained the same or increased after the establishment of school closure, in the absence of a generalized lockdown. This finding suggests that the combination of the two measures is more effective than school closures alone, towards the containment of the clustered spread of the virus between younger individuals. 

Regarding the closure of borders, the impact towards the containment of the epidemic was not reflected on the clustering levels of the sequenced cases, as this measure is mainly effective in controlling the incoming new cases. Observations could be performed for Madrid and Liege where sequence data were available before the establishment and after the end of the measure. The observation that, after the reopening of the borders, there was an increase of the unclustered sequences, which gradually transformed into MTCs specifically in the age group of 20–34 and generally in the age groups referring to adults, suggests that such measures may have a positive impact in controlling the epidemic, while the reopening of the borders can lead to new introductions (unclustered sequences), which are gradually converted into local spreads, reflected in increased MTCs (Figure 3). This finding is in concordance with the studies performed by Wells et al. [71], John et al. [72] and Wilder-Smith et al. [73], suggesting that this measure has a great impact if enacted during the early stages of the epidemic. The estimation of the average time it takes for an incoming case to become domestic and create new MTCs, could be very useful in the context of designing public health interventions as a respond to these introductions. It is very important to rely on molecular tests for the detection of SARS-CoV-2 in the borders after their reopening. Additionally, it is important to highlight the value of continuing molecular testing via real time PCR methods at the border level for Public Health purposes, which should not be ultimately replaced by the recently introduced rapid Ag testing; the isolation of the viral RNA is a prerequisite for PCR testing, providing the necessary material for monitoring the introduction of newly emerged virus variants, like the B.1.1.7, which was recently emerged in the UK. Emerging variants play a pivotal role in the dispersal patterns of cross-borders transmissions, since their virulence and transmissibility might differ [74].

In this study, we relied on data mainly generated during the first pandemic wave, thus some of our inferences may change as more SARS-CoV-2 complete sequences become available. Moreover, because the number of tests for the detection of SARS-CoV-2 that are being held and the proportion of sequenced cases are dissimilar between the geographic locations included in the study, the total number and the profile of the MTCs isolated is not necessarily comparable between these regions. Sequences available on GISAID are unrelated to clinical data, thus the sampling dates do not necessarily reflect the actual infection dates. Other socioeconomic and environmental factors could have also affected the spread of the virus and the formation of MTCs (Figure 2, Table S1). At the same time, other events, such as concerts, parties, political gatherings and sports events could possibly be linked to MTCs, extensive testing, contact tracing, and complete metadata are needed in order to conclude on such associations. However, despite essential differences between countries’ conditions and policies, common trends emerge that prove MTCs are a valuable tool for virus spread surveillance.

Spatiotemporal analysis of MTCs may reflect virus transmission that has not yet been identified through contact tracing, thus cryptic transmissions can be revealed through this process. The investigation of SARS-CoV-2 MTCs via robust bioinformatics pipelines can be a useful tool in order to focus prevention efforts. Routine use of this systematic method in near real-time can automate the detection of SARS-CoV-2 transmission and merits further investigation regarding guidance of Public Health efforts to contain the spread of the pandemic virus.

## Figures and Tables

**Table 1:** Sequences analyzed and MTCs isolated in 10 European geographical regions.

EU Geographical Region	Region’s Specific Sequences, N	Total Sequences in Tree, N	Samples Collection Period	MTCs, N	Sequences in MTCs, N (%)	Number of Sequences in Each MTC, N	Region’s Specific Sequences Enrichment, N (%)	MTC Clades
Munich (DE)	195	8032	2020-03-02 to 2020-05-26	5	51 (26.0)	28 17 12 6 5	19 (67.9) 16 (94.1) 8 (66.7) 4 (66.7) 4 (80.0)	G 32/51 GR 19/51
Vienna (AT)	149	2990	2020-02-26 to 2020-04-14	8	82 (55.0)	44 10 9 9 8 6 6 5	38 (86.4) 9 (90.0) 8 (88.9) 8 (88.9) 6 (75.0) 5 (83.3) 4 (66.7) 4 (80.0)	G 9/82 GR 59/82 S 14/82
Navarra (ES)	109	3646	2020-03-07 to 2020-03-29	3	33 (30.2)	24 11 7	17 (70.8) 11 (100.0) 5 (71.4)	GR 17/33 S 16/33
La Rioja (ES)	256	3779	2020-02-29 to 2020-04-04	9	173 (67.5)	68 51 22 21 16 9 8 6 6	62 (91.2) 35 (68.6) 20 (90.9) 19 (90.5) 15 (93.7) 8 (88.9) 6 (75.0) 4 (66.7) 4 (66.7)	GH 74/173 S 99/173
Lombardy (IT)	412	3333	2020-02-20 to 2020-05-10	14	101 (24.5)	16 12 12 12 11 9 8 8 7 6 6 5 5 5	12 (75.0) 12 (100.0) 11 (91.7) 8 (66.7) 8 (72.7) 8 (88.9) 7 (87.5) 6 (75.0) 6 (85.7) 5 (83.3) 5 (83.3) 5 (100.0) 4 (80.0) 4 (80.0)	G 81/101 GR 20/101
Uusimaa (FI)	227	2979	2020-03-13 to 2020-05-16	7	202 (88.9)	156 66 40 6 6 6 6 5	109 (69.9) 44 (66.7) 31 (77.5) 5 (83.3) 5 (83.3) 4 (66.7) 4 (66.7) 4 (80.0)	G 17/202 GH 185/202
Madrid (ES)	582	4025	2020-02-25 to 2020-08-30	20	163 (28.0)	26 20 19 18 16 14 13 13 9 8 7 6 6 6 6 5 5 5 5 5	17 (65.4) 14 (70.0) 13 (68.4) 13 (72.2) 13 (81.2) 13 (92.9) 11 (84.6) 10 (76.9) 8 (88.9) 7 (87.5) 6 (85.7) 5 (83.3) 5 (83.3) 4 (66.7) 4 (66.7) 4 (80.0) 4 (80.0) 4 (80.0) 4 (80.0) 4 (80.0)	G 108/163 S 50/163 V 5/163
Saint Petersburg (RU)	267	2967	2020-03-13 to 2020-06-16	2	13 (4.8)	11 5	9 (81.8) 4 (80.0)	G 4/13 GR 9/13
Liege (BE)	535	3349	2020-03-05 to 2020-09-25	3	36 (6.7)	27 10 6	21 (77.8) 10 (100.0) 5 (83.3)	G 10/36 GR 26/36
Reykjavik (IS)	601	3044	2020-02-27 to 2020-03-29	6	122 (20.2)	62 47 14 12 9 7	55 (88.7) 31 (66.0) 12 (85.7) 12 (100.0) 6 (66.7) 6 (85.7)	G 62/122 GR 12/122 L 6/122 O 2/122 S 6/122 V 34/122

**Table 2:** Distribution of clustered (belonging to MTCs) and unclustered sequences, according to age and gender of sequenced cases in ten European regions.

		Munich (DE)	Vienna (AT)	Navarra (ES)	La Rioja (ES)	Lombardy (IT)	Uusimaa (FI)	Madrid (ES)	Saint Petersburg (RU)	Liege (BE)	Reykjavik (IS)	Total CL/UN
		Clustered/Unclustered sequences										
AGE	0–19	1/4	5/4	0/1	11/3	1/3	17/3	1/8	0/23	1/15	**18/33**	68/133
	20–34	9/28	8/7	8/6	18/7	5/10	60/4	27/60	0/44	**18/95**	29/113	194/405
	35–49	9/26	17/24	15/21	36/20	17/39	38/2	25/70	**8/49**	5/87	41/157	233/594
	50–64	13/37	29/7	7/26	40/21	19/57	30/4	36/110	3/74	6/70	25/131	243/615
	65–100	19/48	33/24	5/20	27/33	54/184	41/5	70/170	0/47	5/79	8/21	295/693
	Overall	51/143	92/66	35/74	13/74	96/293	186/18	159/418	11/237	35/346	121/455	1033/2440
	Total	194	158	109	206	389	204	577	248	381	576	3473
	Chi’s *p*-value	NS	NS	NS	NS	NS	NS	NS	**	*	*	NS
GENDER	Female	25/54	42/26	21/41	65/29	40/132	90/9	84/206	6/143	11/151	61/214	503/1144
	Male	26/90	40/40	12/34	61/35	56/162	95/9	79/213	7/98	19/163	60/241	512/1262
	Overall	51/144	82/66	33/75	126/64	96/294	185/18	163/419	13/241	30/314	121/455	1015/2406
	Total	195	148	108	190	390	203	582	254	344	576	3421
	Chi’s *p*-value	NS	NS	NS	NS	NS	NS	NS	NS	NS	NS	NS

Note: NS: not significant (*p* > 0.05), *: *p* ≤ 0.05, **: *p* < 0.01. Statistically significant associations in bold.

## Data Availability

Publicly available sequencing data and metadata were downloaded from www.gisaid.org (accessed on 16 October 2020).

## Supplementary Materials

The following are available online at https://www.mdpi.com/2075-1729/11/3/219/s1, Table S1: Demographic and socio-economic factors in 10 European regions, Figure S1: Containment measures in 10 European regions.
