# Supplementary material for: SARS-CoV-2 Molecular Transmission Clusters and Containment Measures in Ten European Regions during the First Pandemic Wave
Source: Life (Basel). 2021 Mar 9;11(3):219. doi: 10.3390/life11030219 (PMC8001481; doi:10.3390/life11030219)
Supplement: Supplementary file 1 [file life-11-00219-s001.pdf]

Table S1. Demographic and socio-economic factors in 10 European regions.

	Munich (DE)	Vienna (AT)	Navarra (ES)	La Rioja (ES)	Lombardy (IT)	Uusimaa (FI)	Madrid (ES)	Saint Petersburg (RU)	Liege (BE)	Reykjavik (IS)
**Sequences in Age groups (%)**										
**(0,19]**	17.97	19.43	20.65	19.26	18.17	22.23	20.21	22.87	22.95	25.94
**(19,34]**	24.11	19.43	15.78	15.09	15.37	21.03	17.25	20.13	19.02	21.05
**(35,49]**	22.19	20.16	23.31	23.43	21.65	21.07	24.71	21.71	19.52	19.46
**(50,64]**	17.97	21.91	20.63	21.31	22.02	18.59	20.11	20.2	20.03	18.37
**(65,100)**	17.77	19.08	19.63	20.91	22.79	17.08	17.72	15.09	18.48	15.19
**Population (N)**	1,538,000	1,930,000	653.85	315,931	10,103,969	1,638,469	6,618,000	5,468,000	197,355	131,136
**Density (people per sq.km)**	4,500	4,574	63	62.8	420	185.7	5,337	3,708.4	2,800	480
**Unemployment rate (%)**	5.9	4.78	9.9	11.5	3.2	6.1	20.45	5.6	5.69	8
**At-risk-of-poverty rate (%)**	16.7	17.5	7.7	12.3	11.1	11	15	13.1	12.8	9
**Human Development Index (HDI)**	0.95	0.92	0.91	0.9	0.9	0.95	0.92	0.89	0.9	0.95
**Annual Gross Domestic Product (GDP) (billion euros)**	189.16	96.4	20.3	8.87	368	91.2	221	51.6	530	19.8
**Life expectancy**	81	82	84.57	83.81	83.2	82	84.99	76.3	80.1	82.55

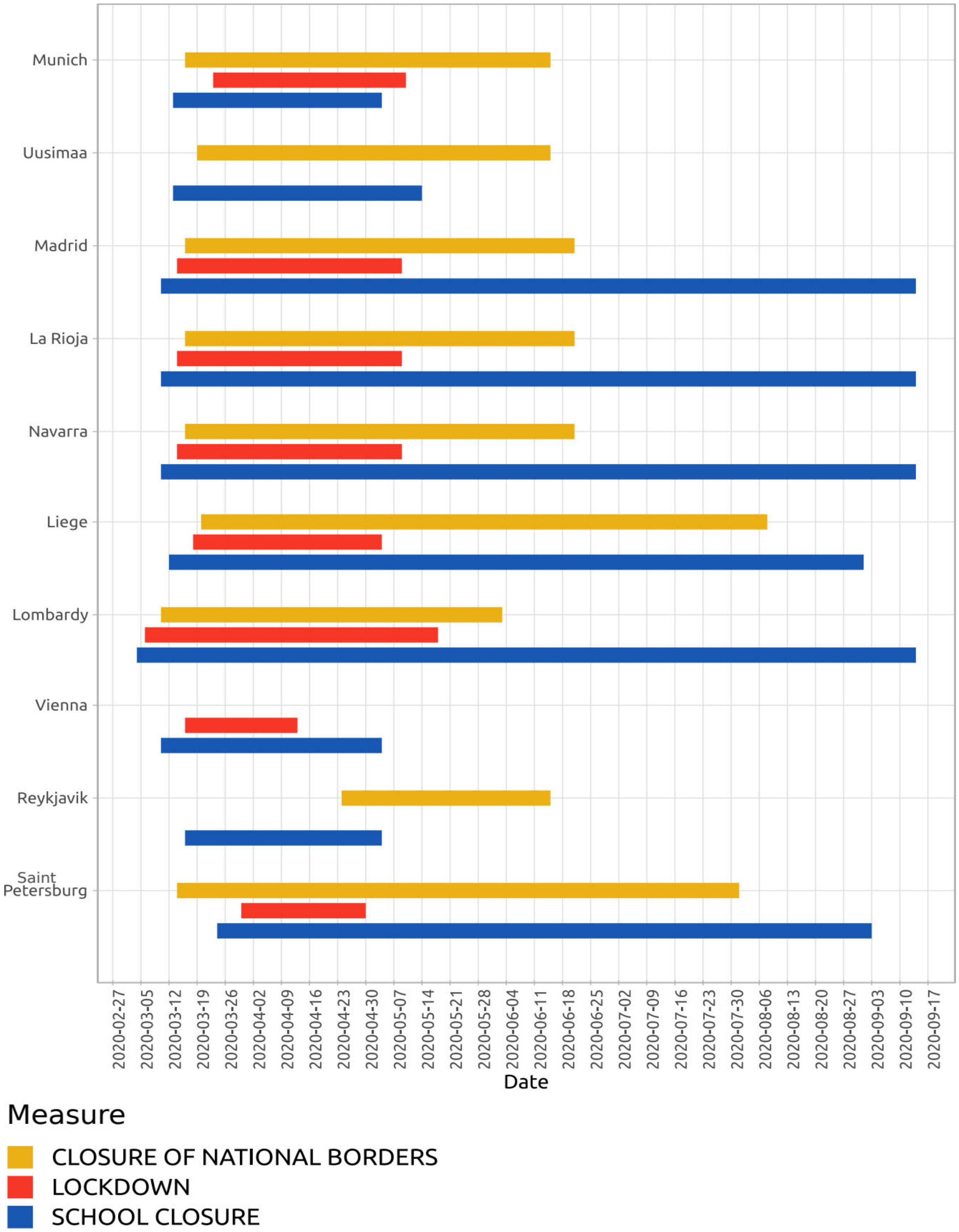

Figure S1. Containment measures in 10 European regions. Gannt chart of the containment measures taken in geographical region. Closure of national borders, lockdowns and closure of schools are presented in orange, red and blue bars respectively.
